# Evaluation of the performance of two neutral oral contrast agents in computed tomography enterography: A randomized controlled trial

**DOI:** 10.1111/1751-2980.12835

**Published:** 2020-01-15

**Authors:** Meng Qi Zheng, Qing Shi Zeng, Yong Quan Yu, Rui Ji, Yue Yue Li, Ming Ming Zhang, Yi Ning Sun, Li Xiang Li, Xiu Li Zuo, Xiao Yun Yang, Yan Qing Li

**Affiliations:** ^1^ Department of Gastroenterology Qilu Hospital of Shandong University Jinan Shandong Province China; ^2^ Department of Laboratory of Translational Gastroenterology Qilu Hospital of Shandong Univerisity Jinan Shandong Province China; ^3^ Department of Robot Engineering Laboratory for Precise Diagnosis and Therapy of GI Tumor Qilu Hospital of Shandong University Jinan Shandong Province China; ^4^ Department of Radiology Qilu Hospital of Shandong University Jinan Shandong Province China; ^5^ Department of Radiology Weihai Central Hospital Weihai Shandong Province China

**Keywords:** computed tomography enterography, mannitol, neutral oral contrast, polyethylene glycols, small intestine

## Abstract

**Objective:**

To compare the performances, tolerability and acceptability of mannitol and polyethylene glycol (PEG) as oral contrast agents in patients undergoing computed tomography enterography (CTE).

**Methods:**

Patients aged 18‐75 years indicated for CTE were randomized to receive either mannitol or PEG as contrast agents. The coronal reconstructed images of each abdominal quadrant were assessed for maximum distention, proportion of distended bowel loops, presence of inhomogeneous contents and visibility of the small bowel wall. Overall subjective imaging quality assessment and patients’ tolerability and acceptability were recorded.

**Results:**

Seventy patients were enrolled and randomized into two groups. In the per‐protocol analysis, no significant differences in imaging quality was found in bowel distention maximum diameter, wall visibility and intestinal homogeneity (all *P* > 0.05). The mean nausea score was lower in the mannitol group (0 [0‐0] vs 1.0 [0‐3.0], *P* < 0.001). Mannitol was superior to PEG in taste (9.0 [8.0‐10.0] vs 7.0 [5.0‐8.0], *P* < 0.001), patients’ willingness to reuse the drug (9.0 [8.0‐10.0] vs 8.0 [7.0‐9.0], *P* = 0.036), satisfaction (9.0 [8.0‐10.0] vs 8.0 [7.0‐9.0], *P* = 0.022) and ease of completion (9.0 [8.0‐9.3] vs 8.0 [6.5‐9.0], *P* = 0.030).

**Conclusions:**

Both mannitol and PEG provided good bowel distention and visualization of the bowel wall. However, mannitol was significantly superior to PEG in patients’ tolerability and acceptability.

## 1 INTRODUCTION

Thanks to the development of imaging technology, computed tomography enterography (CTE) has become one of the first‐line modalities for detecting diseases of the small bowel, particularly inflammatory bowel diseases (IBD).^1^, ^2^, ^3^ Unlike conventional computed tomography (CT) examination, CTE allows the visualization of both small intestinal wall and lumen after ingested a large volume of contrast agents.^4^ Moreover, CTE shows clearly the pathological changes of the small bowel by presenting mural stratification, segmental mural hyperenhancement, increased density of mesenteric fat and engorged vasa recta.^5^, ^6^, ^7^

Quality of a CTE examination depends mainly on adequate intestinal distention and wall visibility, which optimize the resolution of the bowel wall and lumen.^8^ Oral contrast agents used in CTE examinations are considered the key to achieving a satisfactory outcome. In IBD patients the form of mural enhancement is crucial to the diagnosis, especially for Crohn's disease.^9^, ^10^ Neutral oral contrast agents, which are isodense, are preferred to positive oral contrast materials in CTE as these agents improve the conspicuity of bowel mucosal and mural hyperenhancement.^11^, ^12^

Several studies have compared the performances of various neutral contrast agents used in CTE, including water, whole milk, methylcellulose, polyethylene glycol (PEG), mannitol, lactulose and low‐concentration barium.^13^, ^14^, ^15^ Excellent imaging quality combined with a good taste makes mannitol one of the most widely used neutral contrast agents in CTE.^14^, ^16^ However, risks of dehydration and loss of electrolytes, as well as that of producing explosive gas after mannitol ingestion have significantly limited its clinical application.^17^, ^18^ Although no serious adverse reactions have been reported, it remains to be confirmed whether the use of isotonic mannitol as an oral contrast agent in CTE is accompanied by other side effects. PEG has also been recommended as an oral contrast agent for CTE because of its safety; however, certain inherent attributes of PEG may have negative effects on the imaging quality of CTE. Moreover, its poor taste and drainage effect as a volumetric laxative may also affect the imaging quality and patients’ tolerance to the agent.^13^, ^19^, ^20^ As few relevant prospective studies are available,^15^ whether the quality of CTE imaging using PEG as an oral contrast agent was inferior to that using mannitol, despite its safety advantages, remains to be investigated.

The aim of this study was to evaluate prospectively the performances of two neutral oral contrast agents, mannitol and PEG, in CTE, and to assess the image quality and patient's tolerability and acceptability.

## 2 MATERIALS AND METHODS

This study was approved by the Medical Ethics Committee of Qilu Hospital of Shandong University (Jinan, Shandong Province, China) in accordance with the provisions of the Declaration of Helsinki. Written informed consent was obtained from all patients before their enrollment. This study was registered at http://clinicaltrials.gov (no. NCT03495804).

### 2.1 Patients

Adult inpatients aged 18‐75 years undergoing CTE at our hospital from October 2017 to August 2018 were recruited. Exclusion criteria were: (a) aged <18 years or >75 years; (b) with known or suspected bowel obstruction or perforation; (c) severe cardiac dysfunction, severe chronic renal failure (creatinine clearance below 30 mL/min), uncontrolled hypertension (systolic blood pressure >170 mmHg and/or diastolic blood pressure >100 mmHg), or being hemodynamically unstable; (d) with a previous history of colorectal or gastric surgery; (e) with severe IBD or megacolon; (f) with dehydration or dysphagia; (g) pregnancy or lactation; (h) with documented allergy to intravascular contrast agents; and (i) unable to give informed consent. Patients with abnormal CTE imaging related to non‐research factors (ie, intestinal dysplasia) were excluded from per‐protocol (PP) analysis, as were patients who failed to complete the oral preparation as required.

### 2.2 Randomization

The allocation sequences were generated by a computer and encapsulated in sealed, opaque envelopes by an independent investigator. Patients were randomized to receive either of the two neutral oral contrast agents by opening one of these envelopes. The nurses, image technicians, radiologists and analysts were all blinded to the patients' randomization.

### 2.3 Regimens for preparation

The patients were instructed to take PEG (2 L) before the CTE for bowel preparation. They were then randomized to receive 1500 mL oral contrast agent, either mannitol (isotonic mannitol solution diluted with hypertonic mannitol; Baxter, Shanghai, China) or PEG (pineapple‐flavored PEG electrolyte power dissolved to 1500‐mL volume; Wanhe Pharmaceutical, Shenzhen, Guangdong Province, China), in 50 minutes: 1000 mL within the first 30 minutes, 250 mL in the next 10 minutes and the remaining 250 mL in the last 10 minutes. After taking the contrast agent, 20 mg anisodamine hydrochloride was given intramuscularly. Patients were then transferred immediately to the CT room, and CT scan was performed within 10–20 minutes after the injection.

### 2.4 CTE procedure

Plain and contrast‐enhanced triphasic CT scans were performed by using a dual‐source CT (Siemens, Munich, Germany). The scan parameters were as follows: 120 kV, variable mA, rotation time of 0.5 seconds, pitch of 0.6, thickness of 5 mm. An automatic intravenous injection of 60‐100 mL (1.5 mL/kg) contrast material (Ultravist 300 [iopromide]; Bayer Schering Pharma, Berlin, Germany) was administrated via a power injector at a rate of 3.0 mL/s. CT scan was then performed at 30 seconds and 60 seconds, which corresponded to the hepatic arterial phase and the portal venous phase, respectively. The scan ranged from above the diaphragm to the pubic symphysis. Raw data were reconstructed at 1‐mm section widths and a 1‐mm increment.

### 2.5 Assessment of CTE images

The primary outcome was the imaging quality of CTE by using the two neutral oral contrast agents. All the CT findings and coronal reconstruction CT images were obtained and evaluated by a single experienced radiologist (QSZ) in a blinded manner on a computer workstation using a picture archiving and communication system (Impax, AGFA, Mortsel, Belgium). The abdomen was divided into four quadrants using a vertical line through the xiphoid process and a horizontal line through the iliac spine.^13^

Based on prior studies and radiologists' recommendations,^21^, ^22^ intestinal segments of no less than 20 mm in diameter were defined as adequately distended segments. Each quadrant was assessed for the overall distention based on the proportion of adequately distended bowel segments, which was graded on a scale of 1‐4: 1, poor distention (0%‐25%); 2, fair distention (26%‐50%); 3, good distention (51%‐75%); and 4, excellent distention (76%‐100%). The luminal diameter of the most distended bowel loops in each abdominal quadrant was recorded in millimeters. Visibility of the small bowel wall at each quadrant was evaluated according to the visualization of folds and wall of the intestine, which was graded on a scale of 1 (poor) to 4 (excellent). In addition, whether inhomogeneous substances, including solids, semi‐solids and gases, were present in the intestinal lumen at each of the four quadrants were recorded by the observer, which was recorded as “yes” or “no”. The overall evaluation included a judgment on whether the contrast agent had reached the ileocecal part, as well as an overall assessment with a score of 1 (poor) to 4 (excellent), based on the abovementioned criteria.

### 2.6 Assessment of tolerability and acceptability of the patients

The secondary outcomes of the study were the patient's tolerability and acceptability of both neutral oral contrast agents. After completing the CT scan, each patient was interviewed using a questionnaire about their tolerability and acceptability. The patients were asked to rate the degree of nausea, vomiting, diarrhea, abdominal distention and pain during the ingestion of oral contrast regimens on a scale of 0‐10, with 0 being no feeling and 10 being unbearable. They were also asked to rate the taste, ease of completion of the contrast agent ingestion, their own willingness to reuse the contrast agent and their satisfaction with the preparation on a scale of 0‐10, with 0 being unacceptable and 10 being satisfactory. The presence or absence of dizziness, weakness and electrolyte disorders was also documented.

### 2.7 Sample size calculation

The study was designed to be a non‐inferiority trial, which is an experiment to test whether a new drug or therapy is not inferior to an existing positive control drug or therapy. The sample size was calculated based on the average diameter of intestinal distention at all four abdominal quadrants. Assuming a significance level of 0.05, a power of 0.8, a standard deviation of 3 mm and a non‐inferiority margin of 2 mm,^23^ the calculated total sample size required was 58 patients. Considering 15% of the patients might drop out, at least 70 patients should be included.

### 2.8 Statistical analysis

All the statistical analyses were performed by using SPSS for Windows version 21.0 (IBM, Armonk, NY, USA). Continuous variables were presented as mean ± standard deviation and compared using the Student's *t*‐test or the Mann‐Whitney *U*‐test between the two groups. While binary variables were presented as numbers and percentages, and were compared using the χ^2^ test. Multi‐categorical variables were presented as median and interquartile range (IQR) and compared using the Mann‐Whitney U‐test. Intention‐to‐treat (ITT) and PP analyses were used to evaluate the primary outcome. *P* < 0.05 was considered significant for the statistical analyses.

## 3 RESULTS

### 3.1 Patients’ characteristics

From October 2017 to August 2018, 85 eligible inpatients were assessed for inclusion, of whom 15 were excluded due to intestinal obstruction (n = 7), a previous history of colorectal surgery (n = 2), severe constipation (n = 1) and declined to participate in the study (n = 5), respectively. Finally, a total of 70 patients were equally randomized into the mannitol group or the PEG group. Patients’ characteristics are shown in Table 1. There were no significant differences in age, gender distribution, or body mass index between the two groups (all *P* > 0.05).

**Table 1:** Patients’ characteristics

Parameters	Mannitol (N = 35)	PEG (N = 35)	*P* value
Age, y (mean ± SD)	44.9 ± 13.0	44.7 ± 14.4	0.951
Gender, n (%)			0.810
Male	20 (57.1)	19 (54.3)	
Female	15 (42.9)	16 (45.7)	
BMI, kg/m^2^ (mean ± SD)	22.0 ± 3.6	21.5 ± 3.7	0.514

Abbreviations: BMI, body mass index; PEG, polyethylene glycol; SD, standard deviation.

**Figure 1:**
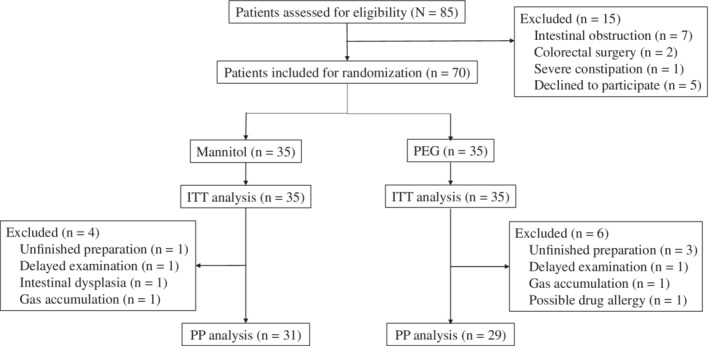
Flowchart of patient inclusion and randomization. ITT, intention‐to‐treat; PEG, polyethylene glycol; PP, per‐protocol

The imaging findings of 60 patients were analyzed in the PP analysis, while 10 patients were excluded due to their failure to finish the examination on time (n = 2), or to take the contrast agent as required (n = 4), intestinal dysplasia (n = 1), possible drug allergy (n = 1) and intestinal gas accumulation (n = 2). The flow diagram of patient enrollment and randomization is shown in Figure 1.

### 3.2 CTE images

The diameters of the most distended bowel loops at the four abdominal quadrants of the mannitol group ranged from 19.0 mm to 26.5 mm, while that in the PEG group ranged 18.3–24.8 mm. In the PP analysis, no significant differences were found in the maximal distention of bowel loops at any of the four abdominal quadrants between the two groups (mannitol vs PEG: left upper [LU]: [21.9 ± 1.7] mm vs [21.7 ± 1.4] mm, *P* = 0.669; right upper [RU]: [21.6 ± 1.5] mm vs [21.8 ± 1.5] mm, *P* = 0.459; left lower [LL]: [21.5 ± 1.0] mm vs [22.1 ± 1.5] mm, *P* = 0.080; right lower [RL]: [22.2 ± 1.7] mm vs [21.8 ± 1.4] mm, *P* = 0.319) (Table 2). Moreover, there were no significant differences between the two groups when assessing the degree of bowel distention using the artificial 4‐point scale (mannitol vs PEG: LU: 2.5 ± 1.1 vs 2.6 ± 1.0, *P* = 0.785; RU: 2.4 ± 0.8 vs 2.5 ± 0.9, *P* = 0.576; LL: 3.2 ± 0.9 vs 3.2 ± 0.7, *P* = 0.815; RL: 2.9 ± 1.0 vs 3.2 ± 0.9, *P* = 0.270).

The mean score for bowel wall visibility in both groups was above the level of good. There were no significant differences at each of the four abdominal quadrants (mannitol vs PEG: LU: 3.4 ± 1.0 vs 3.3 ± 0.8, *P* = 0.642; RU: 3.0 ± 0.8 vs 3.0 ± 0.9, *P* = 0.880; LL: 3.9 ± 0.6 vs 3.8 ± 0.6, *P* = 0.448; RL: 3.5 ± 0.9 vs 3.6 ± 0.8, *P* = 0.741). Additionally, in the ITT analysis, no significant differences were found in these parameters, as shown in Table 2.

**Table 2:** Computed tomography imaging data between the two groups based on intention‐to‐treat (ITT) and per‐protocol (PP) analyses

	ITT analysis			PP analysis		
	Mannitol (n = 35)	PEG (n = 35)	*P* value	Mannitol (n = 31)	PEG (n = 29)	*P* value
Maximum diameter of distention, mm (mean ± SD)						
LU	19.4 ± 7.2	18.0 ± 8.4	0.460	21.9 ± 1.7	21.7 ± 1.4	0.669
RU	19.1 ± 7.1	18.1 ± 8.5	0.597	21.6 ± 1.5	21.8 ± 1.5	0.459
LL	19.1 ± 7.0	18.3 ± 8.6	0.693	21.5 ± 1.0	22.1 ± 1.5	0.080
RL	19.6 ± 7.3	18.0 ± 8.4	0.398	22.2 ± 1.7	21.8 ± 1.4	0.319
Bowel distention score (mean ± SD)						
LU	2.4 ± 1.1	2.3 ± 1.1	0.914	2.5 ± 1.1	2.6 ± 1.0	0.785
RU	2.2 ± 0.9	2.3 ± 1.0	0.903	2.4 ± 0.8	2.5 ± 0.9	0.576
LL	2.9 ± 1.1	2.9 ± 1.1	0.739	3.2 ± 0.9	3.2 ± 0.7	0.815
RL	2.7 ± 1.1	2.8 ± 1.2	0.674	2.9 ± 1.0	3.2 ± 0.9	0.270
Bowel wall visibility (mean ± SD)						
LU	3.1 ± 1.2	2.9 ± 1.2	0.422	3.4 ± 1.0	3.3 ± 0.8	0.642
RU	2.8 ± 1.0	2.7 ± 1.2	0.742	3.0 ± 0.8	3.0 ± 0.9	0.880
LL	3.5 ± 1.1	3.3 ± 1.2	0.342	3.9 ± 0.6	3.8 ± 0.6	0.448
RL	3.2 ± 1.1	3.1 ± 1.2	0.762	3.5 ± 0.9	3.6 ± 0.8	0.741

Abbreviations: LL, left lower; LU, left upper; PEG, polyethylene glycol; RL, right lower; RU, right upper; SD, standard deviation.

Contrast agents reached the cecum in 31 patients in the mannitol group and 29 in the PEG group (*P* = 0.495). By using the ITT analysis, there was no significant difference in the appearance of inhomogeneous substances between the two groups (mannitol vs PEG: 85.7% [120/140] vs 81.4% [114/140], *P* = 0.333). Additionally, the overall assessment of the imaging quality of the two groups showed no significant difference (mannitol vs PEG: 2.6 ± 1.0 vs 2.6 ± 1.0, *P* = 1.000). While based on the PP analysis, both contrast agents reached the cecum in all the patients enrolled. There was no significant difference in the appearance of inhomogeneous substances (mannitol vs PEG: 96.8% [120/124] vs 98.3% [114/116], *P* = 0.457) or the overall assessment of imaging quality (mannitol vs PEG: 2.8 ± 0.8 vs 3.0 ± 0.8, *P* = 0.542) as well.

**Figure 2:**
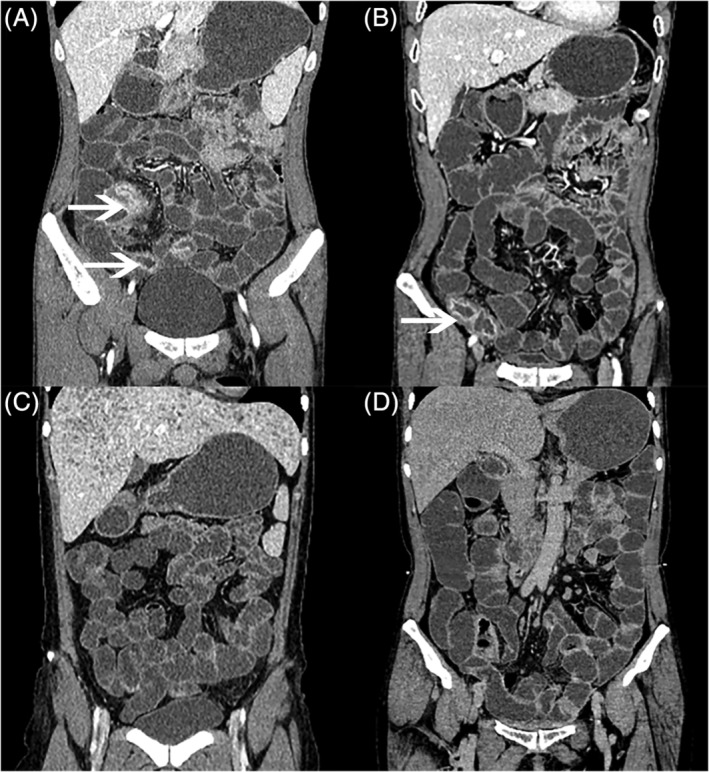
Coronal computed tomography enterography images. A and B, two patients with small bowel diseases; C and D, two patients without intestinal abnormalities. A and C were prepared with mannitol, and B and D were prepared with polyethylene glycol (PEG), respectively. A, B, Both contrast agents clearly depicted mural hyperenhancement, thickening and perienteric inflammatory changes (arrows)

In summary, we found that PEG was statistically not inferior to mannitol in CTE, based on the maximum mean diameter of the distended bowel loops and the imaging quality (Figure 2).

### 3.3 Patients’ tolerability and acceptability of the contrast agents

Adverse events related to the use of contrast agents were analyzed (Table 3). Four (12.9%) patients in the mannitol group reported dizziness after the examination, compared with only one (3.4%) in the PEG group, although there was no statistically significant difference (*P* = 0.185). Three patients each in both groups reported fatigue after the examination (*P* = 0.931). Only one patient in the mannitol group experienced a slight decrease in serum sodium level (134 mmol/L; normal range: 137‐147 mmol/L); while in the PEG group blood electrolyte levels were within the normal range in all the patients (*P* = 0.329).

**Table 3:** Patients’ tolerability to and acceptability of mannitol and polyethylene glycol (PEG) as oral contrast agents by per‐protocol analysis (median [IQR])

	Mannitol (n = 31)	PEG (n = 29)	*P* value
Tolerability (assessed by adverse events)			
Nausea	0 (0‐0)	1.0 (0‐3.0)	<0.001
Vomiting	0 (0‐0)	0 (0‐0)	0.301
Diarrhea	3.0 (2.0‐3.0)	3.0 (2.0‐4.0)	0.244
Abdominal pain	0 (0‐0)	0 (0‐1.5)	0.314
Abdominal distension	2.0 (0‐3.0)	2.0 (0‐3.0)	0.976
Acceptability			
Taste	9.0 (8.0‐10.0)	7.0 (5.0‐8.0)	<0.001
Ease of completion	9.0 (8.0‐9.3)	8.0 (6.5‐9.0)	0.030
Willingness to reuse	9.0 (8.0‐10.0)	8.0 (7.0‐9.0)	0.036
Satisfaction	9.0 (8.0‐10.0)	8.0 (7.0‐9.0)	0.022

Abbreviation: IQR, interquartile range.

The nausea score of the mannitol group was significantly lower than that of the PEG group (0 [0‐0] vs 1.0 [0‐3.0], *P* < 0.001). No significant differences were found between the two groups in the scores of vomiting (0 [0‐0] vs 0 [0‐0], *P* = 0.301), diarrhea (3.0 [2.0‐3.0] vs 3.0 [2.0‐4.0], *P* = 0.244), abdominal distention (2.0 [0‐3.0] vs 2.0 [0‐3.0], *P* = 0.976) and abdominal pain (0 [0‐0] vs 0 [0‐1.5], *P* = 0.314). No serious adverse events were reported in either group.

The score for the taste of mannitol was significantly higher than that of PEG (9.0 [8.0‐10.0] vs 7.0 [5.0‐8.0], *P* < 0.001). The ease of completion score was significantly higher in the mannitol group (9.0 [8.0‐9.3] vs 8.0 [6.5‐9.0], *P* = 0.030). Patients in the mannitol group were significantly more willingly to reuse the drug than those in the PEG group (9.0 [8.0‐10.0] vs 8.0 [7.0‐9.0], *P* = 0.036). Additionally, patients in the mannitol group were significantly more satisfied with their preparation (9.0 [8.0‐10.0] vs 8.0 [7.0‐9.0], *P* = 0.022).

### 3.4 Medical cost of the contrast agents

The cost of mannitol per patient was significantly less than that of PEG. Specifically, for each eligible exam, mannitol cost $1.28 per patient, while PEG cost $8.86 per patient.

## 4 DISCUSSION

Our results showed that both substances were equally effective for imaging quality. However, the patient's tolerability and acceptability of mannitol were significantly superior to those of PEG, and with a lower cost.

This study suggested that the imaging quality using mannitol and PEG as oral contrast agent did not differ significantly with respect to small bowel distention, bowel wall visibility, and intestinal homogeneity. Previous studies have shown that both mannitol and PEG can achieve relatively good imaging quality.^14^, ^19^ Because both mannitol and PEG are isodense that can improve the conspicuity of bowel lumen and wall. Some studies have indicated that mannitol can be fermented by gut bacteria to produce hydrogen and methane gases,^17^, ^18^ which, as inhomogeneous components, may affect the image quality of CTE. In this study, no difference was found in intestinal homogenity between the two groups. This may be because that hydrogen and methane are mainly produced during anaerobic bacterial activity in the human colorectum.^24^ Moreover, the process may be too short to produce detectable gases.

Unexpectedly, in terms of the adverse events, the score for nausea in the PEG group was significantly higher than that of mannitol group, and one patient in the PEG group developed nausea. The score for taste of PEG in our study was significantly lower than that for mannitol, which is consistent with the results of Leduc et al.^13^ Poor taste also raised other issues, as patients in the PEG group were significantly less satisfied with the preparation and showed a significantly lower willingness to reuse the contrast agent than those in the mannitol group.

In this study, the ease of completion score was significantly higher in the mannitol group and the results also showed that the completion rate of the pre‐examination preparation in the PEG group was lower than that of the mannitol group, as three of the four patients who did not complete the prescribed preparation were those who ingested PEG. This may be due to the reason that all the patients used PEG as routine bowel preparation regime before CTE. In the mannitol group, the sequential use of the two agents made the taste of the contrast more distinct; while in the PEG group, repeated use of large volumes of PEG might have led to reduced tolerability.

Because of dehydration and the lack of electrolyte components, water and electrolyte disturbance caused by mannitol is a focus of attention. In our study only one patient experienced slightly decreased sodium level after mannitol ingestion. Possible reasons are as follows: (a) dehydration resulting from mannitol diminished with the decrease in concentration; (b) slight changes in blood electrolyte levels failed to cause corresponding clinical presentations; and (c) the administration of intravenous fluids was the standard practice for the inpatients. This is consistent with the results of other authors,^25^ who found a mild subclinical dehydration with the use of 10% mannitol. The stimulation of mannitol to the intestinal tract is an important factor leading to abdominal cramps and bloating. A comparative study of two oral lavage methods for bowel preparation demonstrated that patients experienced more nausea, cramps, and abdominal pain with 10% mannitol,^26^ but there were no significant differences in abdominal pain and bloating between the two groups in our study. This suggests that low concentrations of mannitol may cause only mild irritation to the gut.

Investigators tend to focus on the effect of contrast agents on imaging quality and have conducted a series of studies on imaging quality by using different contrast agents. However, patient's tolerability and acceptability are also very important, as the large volume of liquid intake is a heavy physical and psychological burden on patients. To our knowledge, there have been no prospective studies comparing both performance and patient's tolerability and acceptability of mannitol and PEG for CTE.

There were several limitations to our study. First, we involved inpatients only in our study. Thus, the efficacy of these two oral contrast agents cannot be generalized to outpatients. Second, patients were not followed up after they completed the questionnaire for subsequent adverse reactions such as diarrhea, leading to the inability to assess the effects on patients' quality of life after the examination. Finally, the patients in our study were not blinded to the agents as the two drugs differed greatly in taste. Therefore, further investigation is needed to overcome these shortcomings.

In conclusion, both mannitol and PEG achieve good imaging quality with respect to the degree of bowel distention, wall visibility and intestinal homogeneity. In addition, the taste of mannitol was significantly superior to that of PEG, which was closely related to its acceptability and made it easier for patients to complete the prescribed preparation. Both mannitol and PEG are relatively safe oral neutral contrast agents. In general, with its good imaging effect and low cost, mannitol is a contrast agent that can be widely used. PEG formulations provide an alternative for some special groups because of its electrolyte composition.

## CONFLICTS OF INTEREST

None.
